# Reproducibility of the 6-minute walk test in lung transplant recipients

**DOI:** 10.1007/s00508-022-02132-w

**Published:** 2022-12-28

**Authors:** Gerold R. Ebenbichler, Gabriella Murakoezy, Julia Kohlmann, Richard Habenicht, Thomas Kienbacher, Peter Jaksch, Patrick Mair, Konrad Hoetzenecker

**Affiliations:** 1 Department of Physical Medicine, Rehabilitation & Occupational Medicine, Medical University of Vienna, General Hospital of Vienna, Währinger Gürtel 18–20, 1090 Vienna, Austria; 2 Department of Thoracic Surgery, Medical University of Vienna, General Hospital of Vienna, Vienna, Austria; 3 Karl-Landsteiner-Institute for Outpatient Rehabilitation Research, Vienna, Austria; 4 Department of Psychology, Harvard University, Cambridge, MA USA

**Keywords:** Lung transplantation, Assessment of activity, Exercise capacity testing, Sensitivity to changes, Rehabilitation

## Abstract

**Purpose:**

There is reason to believe that the favorable measurement properties of the 6‑minute walk test (6MWT) reported for retest reliability and its capability to detect a true change in healthy individuals or persons with chronic respiratory disease may not apply to lung transplant recipients (LuTXr). We therefore investigated retest reliability of the 6MWT and, in addition, made an attempt to explore whether the 6MWT was sensitive enough to detect important changes that occur with postacute rehabilitation in LuTXr after first time LuTX.

**Methods:**

Immediately before postacute rehabilitation, 50 LuTXr completed 6MWT testing twice, separated by 1–2 workdays (retest reliability), and were reassessed after completion of rehabilitation 2 months later (sensitivity to changes). Body function measures and health-related quality of life (HRQoL) assessments were collected at baseline.

**Results:**

Baseline retest 6‑minute walk distance (6MWD) and the age-related predicted walking distance (6MWD%_pred_) scores significantly increased before postacute rehabilitation. The intraclass coefficient of correlation ICC of the 6MWD was 0.93 (95% confidence interval, CI: 0.88–0.96) and its smallest real difference (SRD) 79 m (95% CI: 52;107). Receiver operating curve analyses revealed the rehabilitation associated changes in 6MWD/6MWD%_pred_ to exceed the SRD/SRD% values in a highly accurate way.

**Conclusion:**

The 6MWT overall represents a reliable functional performance tool in LuTXr that is sensitive to detect changes in physical performance as a result of medical postacute rehabilitation.

## Introduction

The 6‑minute walk test (6MWT) measures the distance an individual can walk in 6min and is the most widely used activity based test to assess exercise capacity both in patients with chronic respiratory diseases (CRD) facing LuTX and in lung transplant recipients (LuTXr). It is inexpensive, quickly performed and well tolerated, and requires no special exercise equipment or advanced training [1, 2]. In patients with CRD the 6MWT reproduces an individual’s daily activity, reliably assesses functional exercise capacity, and is sufficiently sensitive to provide information on treatment as well as exercise-based rehabilitation effects [1–3]. In a similar vein, the 6MWT is well correlated with peak oxygen uptake in CRD and it has been shown to be more strongly associated with bodily activities or peak work capacity in CRD than respiratory function tests [4, 5]. Thus, the 6MWT is also used to gauge CRD severity and to predict outcome to either lung transplantation (LuTX) or death in CRD [1, 3, 6, 7], or even survival after LuTX [8]. Whereas the retest reliability and sensitivity to changes of the 6MWT has been well studied in CRD, no such data seem to exist in LuTXr. This is surprising because in LuTXr, the 6MWT is a recommended and widely used measure of paramount importance for monitoring exercise capacity outcomes and as a surrogate measure of impaired functioning and health after LuTX, both in research and clinical practice. Monitoring exercise capacity outcome using the 6MWT would also be strongly endorsed by the World Health Organization (WHO) in its “Rehabilitation 2030” which recommends inclusion of functional measures as part of the routine medical evaluation, and the WHO-ICD 11 is working to establish codes for function [9].

As compared to the symptoms associated with pretransplantation pulmonary failure and the necessity of oxygen supply, patients early after LuTX typically breathe freely without needing additional oxygen. In addition, LuTXr often have a positive perspective on life, as they were lucky to receive an organ and survived LuTX surgery. However, such a positive outlook may be disturbed by unintended complications and side effects of medication, TX organ rejection, bodily deconditioning, and mood disorders among others [10]. These affections can involve any of the organ systems and leave LuTXr impaired in several body functions and limit their activities necessary to participate in important areas of their life. Thus, skilled postacute rehabilitation programs are typically offered to these patients. As most fatal medical complications after LuTX occur within the first 2–3 months after discharge from acute hospital stay, patients are required to conduct rehabilitation programs in close collaboration with the acute hospital transplantation team [11, 12].

In LuTXr, the measurement properties of the 6MWT may differ not only from those in healthy individuals but also from CRD. Thus, the favorable reliability data published for CRD cannot be automatically extended to LuTXr. Indeed, when performing a 6MWT, LuTXr unlike CRD typically do not depend on oxygen supply. Musculoskeletal as well as psychoemotional restrictions are thus the primary limiting factors of the performance levels, if it is assumed that 6MWT scores in LuTXr are less likely to be associated with pulmonary function tests or quality of life measures. In addition, regained independence from oxygen supply in everyday activities of LuTXrs could improve their health-related quality of life (HRQoL) independently from their exercise capacity, lung function tests, and bodily activity levels. In LuTXr, such discrepancies could affect the retest reliability of the 6MWT.

This gap in knowledge led us to investigate whether or not the 6MWT demonstrated retest reliability. In addition, we explored whether the 6MWT as sensitive as to detect important changes that occur with postacute rehabilitation in LuTXr who underwent after first time LuTX.

## Patients, material and methods

### Study setting

This study was conducted at the outpatient department of Physical Medicine, Rehabilitation and Occupational Medicine (PMROM) of the Vienna Medical University Hospital. All LuTXr who participated in this study were tested three times, briefly before discharge from the acute hospital stay (baseline), retested 1–2 workdays later (retest reliability), and after completion of 2 months of postacute rehabilitation (sensitivity to changes). The measurements of the 6MWT were taken on a straight 50 m indoor corridor located within the PMROM department. Three assessors who had received standardized training by a senior clinical specialist, and who have extensive experience in conducting function testing in LuTXr shortly after surgery, performed all the experiments. The training included standardized verbal instructions, standard operating procedures and supervised practice. Assessors performed all tests under guidance by the senior clinical specialist.

### Recruitment

Over the course of a 2.5-year period, a cohort of 30 consecutive non-Austrian LuTXr who had undergone first time LuTX at the department of thoracic surgery, Vienna Medial University were referred for subacute outpatient rehabilitation to the PMROM department and asked to participate in this study. All of these patients agreed. Another 20 sex-matched and age-matched Austrian LuTXr who were awaiting discharge from acute hospital stay at the department of thoracic surgery were also invited and agreed to participate. Thus, a total of 50 (28 females) LuTXr who had undergone first time transplantation were included in the study. This recruitment strategy was applied as foreign citizens who underwent LuTX in Austria were not eligible for inpatient rehabilitation in Austria. By contrast, comprehensive postacute inpatient rehabilitation is strongly recommended and provided for Austrian citizen LuTXr. All participants provided written informed consent and were encouraged to ask questions regarding the study. The ethics committee of the Medical University of Vienna approved the study.

### Sample population

Patients included in the study had to have undergone transplantation of one or two lungs, to be able to stand without support for a minimum of 5 min, and to walk with or without an assisting device for a minimum of 50 m. Exclusion criteria were psychiatric disorders, peripheral neurologic deficits in the lower extremities (except peroneal compression neuropathy), and severe neurologic diseases. Physicians specialized in Physical Medicine and Rehabilitation examined eligible patients. If patients were unable to understand German or English, a translator was made available.

### Interventions

During the entire acute hospital stay, all LuTXr underwent daily rehabilitation. Immediately after discharge from acute hospital care, Austrian citizens (*n* = 20) received 4–6 weeks of inpatient rehabilitation approximately 80 km away from Vienna, whereas foreign LuTXr (*n* = 30) participated in comprehensive outpatient rehabilitation at the PMROM department, which had similar content as inpatient rehabilitation but less supervision. Both the subacute inpatient and outpatient rehabilitation interventions were tailored according to the individual needs of the LuTXr. Relevant components of the rehabilitation program comprised 1) regular cardiopulmonary endurance training, 2) therapeutic exercises to improve muscle and body balance functions, and 3) regular respiratory therapy to improve respiratory muscle function including the breathing pattern and optimizing clearance of sputum. Nutritional and psychological counselling was mandatory for inpatients and on demand for outpatients.

### Procedures

#### 6MWT

Participants walked at a self-paced speed as far as possible along a 50 m indoor straight course. They were encouraged to attempt to cover as much ground as possible in the allotted time interval. The assessors encouraged the participants with standardized statements. Participants were allowed to stop and rest if exhausted but were encouraged to continue as soon as they felt able to do so. The technical procedures followed the recommendations for the 6MWT published by the American Thoracic Society [13] except for the trail length which was 50 m instead of 30 m. A trail length longer than 30 m was found not to effect gait distance in a significantly way [14]. Time elapsed was measured at every 100 m interval. At the end of the test, participants rated their degree of exhaustion on an 11-point Borg scale (0 = no exhaustion, 10 = most severe exhaustion imaginable) [15]. As the 6MWT in LuTXr is frequently limited by activity-related, fatigue-induced sensory perceptions in the legs (intermittent, claudication-like pain), participants further rated the intensity of their pain at its worst on an 11-point VAS pain scale (0 = no pain, 10 = most severe pain imaginable).

#### Functional measures

Maximum hand muscle grip strength (Jamar®, JLW Instruments, Chicago, IL, USA) [16], five times chair rise time (CR), standard spirometry data (MasterScreen Body, Jäger, Germany), body mass index, the European quality of life 5D (EQ-5D-3L) instrument [17] and the short form 36 test (SF-36) [18] were collected at baseline.

### Data processing

The distance walked was assessed in meters (6MWD) and its predicted walking distance (6MWD%_pred_) was calculated for males and females separately according to published reference equations [19]. From the 100 m split distances, walking speed intervals were estimated for each minute of the 6MWT and the respective dynamics determined by calculating the percentage change in speed for each consecutive minute covered compared to the first minute, starting from the second minute [(speed min_n_ − speed min_1_/speedmin_1_) × 100%].

### Sample size

With a minimal expected intraclass correlation coefficient (ICC) of 0.85 and a hypothesis that the present findings would be consistent with a minimum ICC of 0.9, a minimum sample size of 38 individuals was required to achieve a level of significance of 0.5 and a power of 0.8 (β = 0.2). Because 20% of participants usually refuse a second testing, we sought a sample size of 50.

### Statistical analyses

All statistical analyses were performed using the R environment for statistical computing [20]. Distribution of the 6MWT components were assessed and appropriate reliability indices were compiled using previously suggested [21, 22] data inspection procedures. The following aspects were explored for the variables obtained from the first 2 test days:

1. The systematic bias, by calculating the differences of the means and accompanying 95% confidence intervals (95% CI).
2. Precision of measurements, by calculating the standard error of measurement (SEM) estimated as the square root of the mean square error term from the 2‑way ANOVA [22], the smallest real difference (SRD = 1.96 × √2 × SEM) derived from the 2 test days and its respective smallest real difference in absolute values (SRD) or relative to its mean in % (SRD%) [23].
3. Bland-Altman plots [24].
4. Relative reproducibility, using the intraclass correlation coefficient (ICC2,1), which considers systematic changes [22].

### Sensitivity of the 6MWT to rehabilitation

Paired t‑tests calculated the 6MWT score changes between baseline, the first re-test and completion of rehabilitation. Receiver operating analyses (ROC) served to explore the ability of the 6MWT scores to detect changes that occur with rehabilitation in an accurate way. Responders and non-responders were classified as follows:

1. those LuTXr whose changes in 6MWT scores from baseline (in a second analysis form baseline retest) to the end of rehabilitation exceeded the calculated SEM and SEM%, indicative of the smallest detectable change,
2. those LuTXr whose changes in 6MWT scores from baseline and baseline retest to the end of rehabilitation exceeded the calculated SRD and SRD%, indicative of a true change.

## Results

Of the 50 LuTXr enrolled 44 completed retesting, 1 work day after the first test. Two of these patients declined to participate due to their deteriorated health, which was unrelated to the testing on the second test day, and the others decided to withdraw for personal reasons. At the end of rehabilitation a total of 11 patients (5 of these LuTXr were also not available for baseline retesting before rehabilitation) did not participate in the follow-up assessment. Of these, four patients were unable to reach the clinic as they had already returned to their home countries, and another seven patients refused testing for non-medical reasons. Baseline 6MWT scores did not differ between LuTXr who dropped out of the study at follow-up and those who completed the study. All tests were performed without any side effects. Demographic variables are provided in Table 1 and baseline characteristics in Table 2.

**Table 1:** Demographic and lung function parameters at baseline (test day 1), briefly before discharge from the acute hospital stay

	LuTXr sample n/mean (SD)
*Number of patients*	50
*Age (in years)*	38.4 (12.8)
*Body mass index (kg/cm*^*2*^*)*	19.57 (3.9)
*Disease causing LuTX (n)*	
Cystic fibrosis	26
Chronic pulmonary disease	7
Chronic pulmonary hypertension	8
Others	9
*Medication number of patients (in mg)*	
Glucocorticoid (prednisolone) (*n* = 50)	36.22 (48.08)
Tacrolimus (*n* = 48)	11.56 (5.92)
Cyclosporine (*n* = 1)	400.00 (–)
Mycophenolate mofetil (*n* = 26)	697 (1122)
*Time elapsed from*	
Transplantation to first test (days)	31.4 (33.1)
Duration at ICU (days)	16.1 (30.1)
Baseline to 1st retest (days)	1.5 (1.4)
Baseline to end of rehabilitation (days)	77.4 (35.9)
*Lung function related variables (discharge from acute hospital):*	
Lung vital capacity (l)	1.9 (0.7)
Lung vital capacity (% predicted)	42.9 (12.7)
FEV1s (l)	1.7 (0.6)
FEV %	45.4 (13.0)
MEF50 (l)	2.9 (1.3)
MEF50 (%)	64.1 (28.8)

*FEV1s* forced expiration volume in 1 s, *MEF50* mean expiratory flow 50%

**Table 2:** Baseline characteristics of the study participants

			All participants	Age < 39 years	Age ≥ 39 years	Age difference
		*n*	Mean (SD)	Mean (SD)	Mean (SD)	*p*-value
*6MWT*	*6MWD (m)*	50	372.1 (105.2)	370.9 (120.4)	373.4 (89.9)	0.8
	*Age related norm %*	50	52.7 (14.3)	47.1 (14.3)	58.2 (12.2)	0.004
	*Leg pain end of test*^a^ *(VAS: range 0–10)*	22	6.6 (1.4)	7.0 (1.1)	6.1 (1.7)	0.2
	*% of patients with leg pain during 6MWT*	43	64%	68%	60%	–
	*Perceived exhaustion (BORG: range 0–10)*	50	5.9 (1.8)	5.9 (2.1)	6.0 (1.6)	0.9
*HR*	*Base (HRR%)*	49	134.7 (54.6)	117.8 (42.4)	150.9 (60.6)	0.04
	*Peak (HRR%)*	47	159.7 (54.9)	142.9 (41.7)	177.3 (62.1)	0.05
	*1* *min after (HRR%)*	39	146.6 (55.5)	131.1 (45.2)	162.9 (61.6)	0.07
*Other*	*Grip strength (kg)*	50	16.5 (13.0)	12.9 (12.2)	20.1 (13.0)	0.04
	*EQ5D score*	50	0.74 (0.20)	0.75 (0.20)	0.74 (0.21)	0.9
	*Chair rise time*^b^ *(s)*	48	29.5 (21.3)	34.0 (21.8)	25.4 (20.3)	0.2
	*% of patients where chair rise test possible*	50	70%	64%	76%	–
	*SF-36 physical functioning*	48	43.7 (25.7)	49.0 (26.4)	38.3 (24.3)	0.1
	*SF-36 social functioning*	48	47.7 (31.1)	46.4 (33.9)	49.0 (28.5)	0.8
	*SF-36 mental score*	44	39.5 (10.4)	40.1 (10.8)	38.9 (9.9)	0.7

*6MWT* 6-Minute walk test, *6MWD* 6-Minute walk distance, *HR* Heart rate, *HRR* Heart rate reserve, *VAS* Visual analogue scale, *BORG* BORG rating of perceived exhaustion

^a^Patients without pain (pain = 0) excluded

^b^Chair rise time scores exceeding 60 s were classified as “impossible to complete the task”

**Fig. 1:**
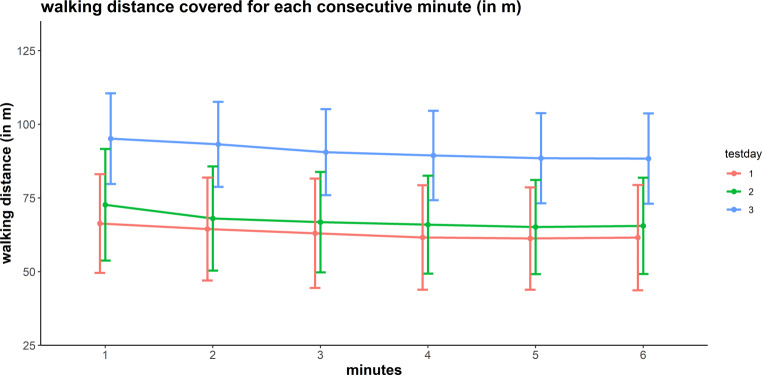
6MWT profile of the first, second and third tests

Examination of age-specific differences in baseline characteristics revealed no significant differences for the variables assessed at baseline, except for the 6MWD_pred_ scores, which were lower in younger than in older LuTXr (Table 2). On the first of the 2 test days, none of the participants exceeded the lower cut-off value (82%) of the 6MWD predicted scores, whereas 16 LuTXr exceeded this value at the end of rehabilitation. The 6MWD significantly increased between baseline and retest, paralleled by an increase in perceived exertion sores at the end of the test (Table 3, Fig. 1). This increase was significant in LuTXr who covered more than 300 m.

**Table 3:** Results of reliability testing of the 6MWT outcome variables 6MWD (m) and 6MWD_pred_ and changes as at end of rehabilitation

		Assessment			Reliability					Change upon completion of rehabilitation	
		1st	2nd	3rd	Difference 2nd to 1st assessment					Difference 3rd to 1st assessment	
		Mean (SD)	Mean (SD)	Mean (SD)	Mean (95% CI)	ICC (95%CI)	SEM (SEM%)	SRD (SRD%)	*p*-val	Mean (95% CI)	*p*-val
*All patients*	*6MWD*	372.1 (105.2)	397.1 (111.1)	538.3 (95.7)	27.0 (14.6, 39.4)	0.93 (0.88, 0.96)	28.5 (7.4)	79.0 (20.6)	< 0.001	174.2 (150.9, 197.5)	< 0.001
	*&MWD age related norm %*	52.7 (14.3)	55.9 (15.4)	77.6 (14.9)	4.0 (2.2, 5.7)	0.93 (0.88, 0.96)	3.9 (7.4)	11.0 (20.4)	< 0.001	25.98 (22.6, 29.4)	< 0.001
	*Perceived exhaustion (BORG: range 0–10)*	5.9 (1.8)	6.9 (1.8)	5.4 (2.0)	0.9 (0.3, 1.5)	0.45 (0.18, 0.66)	1.3 (20.8)	3.7 (57.7)	0.002	−0.7 (−1.3, 0.0)	0.04
	*Leg pain end of test *^*1*^* (VAS: range 0–10)*	6.6 (3.4)	6.9 (3.8)	5.4 (3.1)	0.0 (−1.2, 1.1)	0.17 (−0.34, 0.60)	1.5 (22.3)	4.2 (61.7)	0.9	−1.41 (−2.4, −0.4)	0.009
**Age specific subgroup analyses**											
*Age* *≤* *39*	*6MWD*	370.9 (120.4)	387.5 (124.6)	540.2 (106.0)	20.0 (−0.2, 40.2)	0.93 (0.84, 0.97)	33.0 (8.7)	91.6 (24.2)	0.052	185.9 (145.2, 226.6)	< 0.001
	*6MWD age related norm %*	47.1 (14.3)	49.1 (15.1)	69.1 (11.9)	2.7 (0.1, 5.4)	0.92 (0.81, 0.96)	4.3 (9.0)	12.0 (24.9)	0.044	24.71 (19.3, 30.1)	< 0.001
	*Perceived exhaustion (BORG: range 0–10)*	5.9 (2.1)	6.9 (2.0)	5.2 (2.2)	0.9 (−0.1, 1.8)	0.40 (−0.01, 0.69)	1.6 (25.12)	4.4 (69.6)	0.1	−1.0 (−2.1, 0.08)	0.07
	*Leg pain end of test *^*1*^* (VAS: range 0–10)*	7.0 (3.6)	7.0 (3.8)	6.0 (3.4)	−0.3 (−1.7, 1.2)	0.26 (−0.41, 0.74)	1.4 (19.9)	3.8 (55.1)	0.9	−0.9 (−2.8, 1.0)	0.4
*Age* *>* *39*	*6MWD*	373.4 (89.9)	408.0 (95.3)	536.6 (87.4)	35.1 (20.7, 49.4)	0.95 (0.88, 0.98)	21.7 (5.6)	60.0 (15.4)	< 0.001	163.1 (136.1, 190.1)	< 0.001
	*6MWD age related norm %*	58.2 (12.2)	63.8 (11.9)	85.7 (13.0)	5.4 (3.2, 7.6)	0.93 (0.83, 0.97)	3.4 (5.6)	9.3 (15.4)	< 0.001	27.2 (22.7, 31.7)	< 0.001
	*Perceived exhaustion (BORG: range 0–10)*	6.0 (1.6)	7.0 (1.5)	5.7 (1.7)	0.9 (0.3, 1.6)	0.57 (0.18, 0.80)	1.0 (15.3)	2.7 (42.3)	0.02	−0.4 (−1.2, 0.5)	0.4
	*Leg pain end of test *^*1*^* (VAS: range 0–10)*	6.1 (3.3)	6.8 (3.6)	5.0 (2.9)	0.3 (−2.3, 3.0)	0.03 (−0.74, 0.77)	1.8 (27.8)	4.9 (77.0)	0.8	−1.8 (−3.3, −0.4)	0.03

Please note that this table also includes a subsequent analysis of younger and older than 39 years old. The cut age was calculated by median split method.

*6MWD* 6-Minutes walk distance; *VAS* Visual analogue scale; *BORG* BORG rating of perceived exhaustion

### Reliability

Measurement error among all the participants amounted to 34 m for the 6MWD and 3.9% for the 6MWD%_pred_. Both the SRD of the 6MWD and the 6MWD%_pred_ calculated for the entire group were 79 m (mean: 79 m) and 11%, respectively (Table 3). The ICCs achieved excellent between test days consistency of the individual 6MWD scores ranking (Table 3). Subsequent age-specific sub-analyses revealed numerically higher SEM and corresponding SRD values for both 6MWT scores in younger as compared to older LuTXr (Table 3).

**Fig. 2:**
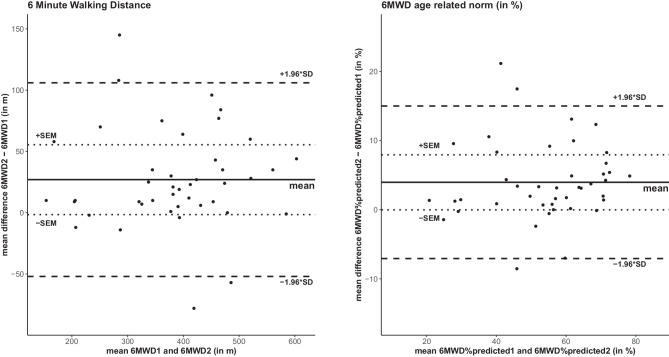
Bland Altmann plots for the difference between two 6MWTs at the initial assessments. Note that 46 LuTXr completed the retest on the second test day before postacute rehabilitation started. *Solid line* mean difference test 1 and 2; *dashed line* coefficient of variability (±1.96 * SD); *dotted line*: measurement error. *6MWD* distance walked in the 6MWT, *6MWD%*_*pred*_ the predicted walking distance (6MWD), *6MWD1 and 6MWD2* first and second 6MWT

Bland-Altman plots indicated a systematic bias between the two baseline measurements, and the magnitude of agreement appeared to decrease when 6MWD increased. The 95% CI limit of agreement of the 6MWD and the 6MWD%_pred_ were between 52–106 m and 7–15%, respectively (Fig. 2).

### Sensitivity to changes

**Fig. 3:**
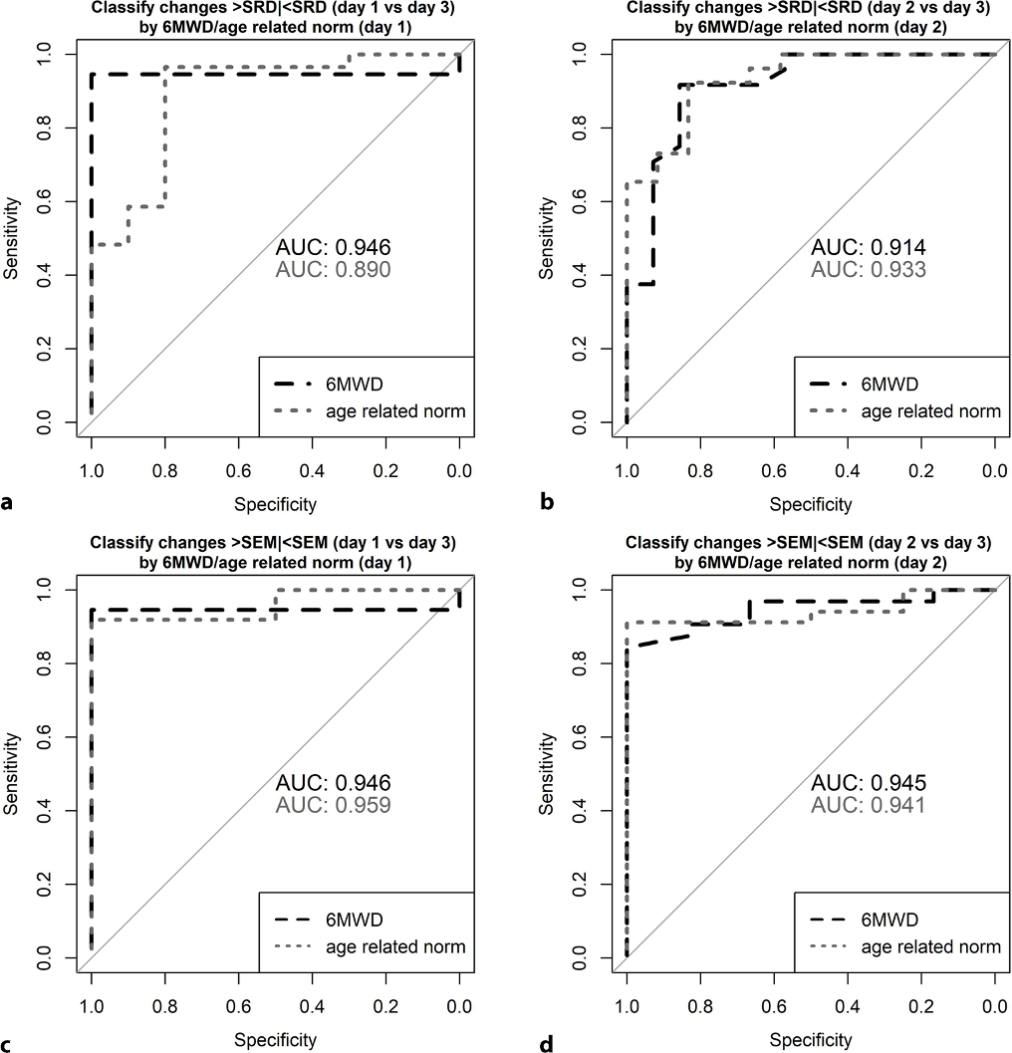
The receiver operating curves (ROC) explore the sensitivity of the 6MWT to detect a true change (changes in 6MWT scores upon completion of rehabilitation > SRD (smallest real difference)) and a minimum change (changes in 6MWT scores upon completion of rehabilitation > SEM) in 6MWD or 6MWD%_pred._ Note that the ROC analyses were performed twice: the first one considers the rehabilitation associated changes as of baseline testing (**a**,**c**), the second one as of baseline retesting (**b**,**d**). *6MWT* 6-Minute walk test; *6MWD* 6-Minute walk distance. *SEM* Standard error of measurement; AUC Area under the curve derived from receiver operating analyses (ROC).

When compared to baseline, the 6MWD significantly increased by 174.2 m (95% CI: 150–198) and its 6MWD%_pred_ score by 26% (95% CI: 23–29) upon completion of rehabilitation. ROC analyses revealed the 6MWT scores as highly accurate in detecting both a minimum and a true change because of postacute rehabilitation. This was true for changes with rehabilitation as assessed from baseline or baseline retesting. The respective AUC values exceeded 0.89 and are provided in Fig. 3.

### Discussion

This study’s major findings revealed: 1) a systematic change in the mean between test and retest; 2) ICC values indicating excellent relative reliability; and 3) SRD values that allowed detection in 6MWT score changes observed at the end of postacute rehabilitation.

A previous systematic review found the 6MWD to be a reliable measure in CRD with respective ICCs ranging from 0.72 to 0.99 [1]. Data from our study in LuTXr suggest that the 6MWD and 6MWD%_pred_ scores are as reliable as in CRD. In this study, the mean ICCs of the 6MWD scores and their respective 95% confidence intervals exceeded 0.89 and are proof of a high degree of consistency and agreement in LuTXr shortly after LuTX surgery, if the 6MWT is repeated on a second test day.

Despite the fact that LuTXr are familiar with the 6MWT, we observed an improvement in 6MWD of a mean of 27 m (95% CI: 14.6–39.4) during the baseline retest. This is surprising, as such changes in the mean are typically observed when patients are either unfamiliar with the test or have not performed it for a sufficiently long period of time. By comparison, in novice test subjects an improvement in 6MWD on retest was found to be 19 m in patients with pulmonary hypertension [25], and 26.3 m amongst COPD patients [1]. Derived from the SEM, the smallest clinical important difference (CID) for the 6MWD in LuTXr on an individual level would be 28.5 m. This estimate, which is similar to the CID scores determined for CRD (29–34 m [26], 30 m [1], 29–42 m [27]), is close to the changes in the mean observed on retesting in LuTX. This result strongly points to the value of repeating the test on a second occasion in LuTXr shortly after transplantation, regardless of their previous experience with the 6MWT. This view could be further corroborated by the parallel increase in perceived physiological effort with the improved 6MWD scores in LuTXr at baseline reresting, a result which was not observed in research amongst CRD patients [25]. Thus, LuTXr seem motivated to perform higher on a 6MWT retest occasion despite the standardization of the verbal encouragement provided. Greater confidence in the “new” lung and more appropriate subjective interpretation of shortness of breath because of deconditioning rather than need of oxygen supply, and, potentially, a more positive experience with the testing on the first test day may all serve as explanations. This may be particularly true for older as compared to younger LuTXr, as they were found to have more pronounced learning effects in our secondary, age-specific sub-analysis. Consequently, the second test would better reflect the LuTXr’s true exercise capacity. It is unlikely that a standardized psychoemotional intervention could minimize a potential learning effect in LuTXr, particularly in day-to-day clinical practice, where repeating the 6MWT on a second test day is typically not feasible. It is worth noting that, like in COPD, a learning effect in LuTX patients who covered less than 300 m was absent. Amongst these individuals, physical limitations of exercise performance likely outweighed the psychoemotional ones.

In this study we measured intermittent claudication-like leg pain during walking, which was observed in 64% of our LuTXr when tested at baseline. This type of pain typically occurred in the shanks and/or thighs after 2–3 min of walking and its intensity was rated to be moderate to severe at the end of the 6MWT. It was notable that none of the LuTXr suffered from peripheral occlusive disease. Nevertheless, the LuTXr walking speed was well maintained throughout the test, as illustrated in Fig. 1. It therefore seems likely that claudication-like leg pain does not affect the 6MWD scores in a relevant way. Although underlying mechanisms causing this activity-related pain in LuTXr remain widely unknown, relative overuse of the weight-bearing working muscles during walking best explains this type of pain. Therefore, overly accumulating metabolites associated with excessive muscle fatigue would elicit pain by activating type III and IV nerve endings located within muscles and surrounding tissue. This could be due to disuse-related, impaired muscle fiber metabolic capability (of both type I and II fibers) [28] and/or a relative lack of oxygen supply to the working muscle. A lack of oxygen supply would be associated with a presumptive loss in muscle fiber capillary density, resulting in impaired matching between oxygen delivery and oxidative metabolism [29, 30]. In LuTXr, such disuse associated muscle metabolic impairments during exercise could further be aggravated by adverse effects of immunosuppressive medication to mitochondria and/or to the expression of type II muscle fibers [31–33].

Perceived exhaustion is widely measured at the end of the 6MWT as it provides an estimate for the degree of deconditioning of LuTXr. Instead of 6MWT BORG scale measures of dyspnea in chronic lung disease, we collected perceived exhaustion BORG scale ratings as this variable seems more appropriate in LuTXr early after LuTX. In LuTXr oxygen saturation levels were within normal ranges (> 90%) throughout the test. Walking speed in the 6MWT is self-adjusted and the motivation to walk for 6 min as far as possible is known to vary considerably between patients. Considering all this, assessments of BORG ratings may aid in validating the 6MWT scores obtained, and furthermore appear to be particularly useful if 6MWT scores represent normal or close to normal values. In such cases, the degree of exhaustion would allow estimation of how vigorously the test subject performed the 6MWT. It is important to note that the variability of subjective BORG scale ratings is higher than that observed from objective measures [34]. Thus, these scores may not yet qualify for inclusion in a complex 6MWT score, unless fully validated.

For clinicians it is of utmost importance to know whether the 6MWT is sensitive to interventions. This study revealed that the changes in distance walked upon completion of rehabilitation clearly exceeded the SRD 79 m. Considering our favorable findings from ROC analyses, the SRD appears small enough to allow identifying improvements in 6MWD at the end of postacute rehabilitation or therapeutic exercise interventions within the first year of rehabilitation [35–37]. Such favorable AUC values seemed not to be affected even when the 11 participants lost to follow-up upon completion of rehabilitation were considered. This observation is indirectly supported by both a) the non-significant differences in baseline 6MWT scores between those who completed the study and those lost to follow-up, and b) the reports that neither a low functioning and health states nor death was the main reason of not showing up. Conversely, the tight schedule of the regular comprehensive medical assessments at the thoracic surgery department along with the burden of travelling very long distances of several hundred kilometers on one day were primary reasons for not participating in follow-up assessment. However, the 6MWD may not be sufficiently sensitive if the improvements to inpatient rehabilitation need to be identified 5 years after LuTX [38]. Of note, in this study the majority of LuTXr had already suffered from bronchiolitis obliterans. It is worth noticing that our study also included young patients. Because their postoperative 6MWD%_pred_ (but not absolute 6MWD) scores were significantly lower at baseline, they might improve faster and to a greater extent after surgery because of their stronger physiological reserve capacity [39]. However, considering the smaller SEM and SRD values observed from our older LuTXr group, responses to postacute rehabilitation would likely be equally identified with the 6MWD%_pred_ scores in younger and older LuTXr.

### Limitations

We studied retest reliability in LuTXr when discharged from the acute hospital after LuTX. Although the mean 6MWD score values after discharge from the acute hospital stay are smaller than in a later phase of LuTX, the dispersion of scores may be expected to be similar or even broader. This would boost the ICCs observed without affecting the 6MWD’s sensitivity to identify relevant changes. Additionally, when reliability is studied in an early phase after LuTX, a learning effect might become more overt despite an individual’s familiarity with the test. This interpretation suggests that our results, which are related to the reliability of the 6MWT in postacute phase after LuTXr, cannot be extended to later phase LuTXr. However, another study that assessed the mean and SD of the 6MWD 1 year [40] and 5 years after LuTX [38] suggests that the reliability of the 6MWD would not change in a relevant way if investigated in a later phase after LuTX. In these studies, the mean 6MWD scores (523 m at 1 year [40] and 490 m at 5 years[38] after LuTX) were clearly higher than in ours, but the dispersion of scores as expressed by their SD was comparable. As learning effects were found to be present and of a similar extent as early as 3 months after a 6MWT in CRD [41], this evidence suggests that the metric properties of the 6MWT could be administered to LuTXr at any phase after LuTX. However, future studies will have to clarify this point.

## Conclusion

This study’s findings suggest that the 6MWT is overall reliable in LuTXr and enables detection of changes in bodily reconditioning as a result of planned medical rehabilitation interventions in the first year after LuTX. Retesting at baseline is recommended particularly in higher performers.
